# Dual Inhibition of PARP and Akt Induces Metabolic Collapse and Apoptosis in Breast Cancer Cells

**DOI:** 10.3390/cancers17233828

**Published:** 2025-11-29

**Authors:** Nasreldeen Mohamed Karshom Adam, Eszter Vámos, Hamid Ahmadi, Geofrey Ouma Maloba, Arshi Arshi, Ferenc Gallyas Junior

**Affiliations:** 1 Department of Biochemistry and Medical Chemistry, Medical School, University of Pécs, 7624 Pécs, Hungary; karshom.nasreldeen@pte.hu (N.M.K.A.); eszter.vamos@aok.pte.hu (E.V.); maloba.geofrey.ke@gmail.com (G.O.M.); arshi@pte.hu (A.A.); 2 Department of Medical Biology, Medical School, University of Pécs, 7624 Pécs, Hungary; hamid_ahm1986@yahoo.com

**Keywords:** ATP production, mitochondrial respiration, glycolysis, apoptosis, reactive oxygen species, TNBC, MCF7

## Abstract

Background: Breast cancer is the most prevalent cancer among women worldwide, and therapeutic resistance represents a major clinical challenge. Mitochondria are key regulators of cancer metabolism, redox homeostasis, and apoptosis, making them potential therapeutic targets. Aim: This study aimed to evaluate the effects of combined Akt and PARP inhibition on mitochondrial metabolic function, energy production, and apoptosis in breast cancer cells. Methodology: The SRB assay was used to compare the viability of MDA-MB-231 and MCF7 cells. A colony formation assay was conducted to assess the capacity of individual cells to develop colonies, and ROS production was quantified using DHR123. Flow cytometric analysis was performed to evaluate cell death, and the Seahorse Mito stress test was used to measure ATP production and essential mitochondrial parameters. Results: The combination of Akt and PARP inhibitors impaired oxidative phosphorylation without inducing a compensatory shift to glycolysis, leading to reduced ATP production, increased ROS generation, and apoptotic cell death in breast cancer cells compared to monotherapy. Conclusions and Recommendations: These findings indicate that the combination of olaparib and capivasterib is a promising therapeutic strategy for breast cancer. Furthermore, evaluation of in vivo toxicity and antitumor effectiveness is essential to validate its potential.

## Simple Summary

Breast cancer remains one of the leading causes of death among women globally, demonstrating complex molecular mechanisms and poor response to existing therapies. Despite the development of chemotherapeutic options, chemotherapy resistance, especially in aggressive subtypes such as triple-negative breast cancer, is a substantial clinical concern. Identifying the potential factors that induce resistance in breast cancer is a promising therapeutic strategy to overcome drug resistance. Here, we demonstrated the cancer-inhibiting activity of a combination of PARP and Akt inhibitors, as well as their effects on mitochondrial metabolic function and overall energy outcomes. We found that the combination therapy impaired oxidative phosphorylation without inducing compensatory glycolytic response, leading to reduced ATP production, increased ROS generation, and apoptotic cell death. A novel combination treatment arising from the combination effects of olaparib and capivasertib in a breast cancer cell lines was more effective than individual treatments in MDA-MB-231 and MCF7 cells.

## 1. Introduction

According to a report by the World Health Organization in 2022, breast cancer (BC) is the most prominent cancer subtype among women worldwide. Approximately 2.3 million new cases of breast cancer patients are reported globally each year [1,2,3].

The improvement in targeted therapeutic plans, such as cyclin-dependent kinase 4 and 6 (CDK4/6) inhibitors, phosphatidylinositol 3-kinase (PI3K) inhibitors, and selective estrogen receptor degraders (SERDs), has significantly expanded the treatment options for patients with estrogen or progesterone receptors and human epidermal growth factor receptor 2 (HR+/HER2-) breast cancer, leading to improved outcomes and a better understanding of the underlying molecular drivers of this disease [4]. Poly (ADP-ribose) polymerase (PARP) inhibitors block the repair of single-stranded breaks (ssDNA), leading to their accumulation [5]. This is particularly effective in neoplasms with defects in homologous recombination repair, including those with breast cancer gene 1 (*BRCA1*) or breast cancer gene 2 (*BRCA2*) mutations, representing a hallmark example of synthetic lethality and a major therapeutic advance for targeted Triple-Negative Breast Cancer (TNBC) [6].

Olaparib is a targeted cancer therapy that is a PARP inhibitor, designed to interfere with DNA repair mechanisms in cancer cells [7]. It was first officially authorized by the Food and Drug Administration (FDA) in 2018, and the treatment received full regulatory approval for patients with deleterious or potentially deleterious germline *BRCA*-mutated (g*BRCA*m) and human epidermal growth factor receptor 2-negative (HER2-negative) disseminated breast cancer who received chemotherapy before surgery (neoadjuvant), after surgery (adjuvant), or in a metastatic disease context. Olaparib has demonstrated significant benefits in terms of improvement and survival, while maintaining a relatively favorable safety profile, with patients reporting better health-related quality of life during treatment with olaparib than with chemotherapy [8].

The phosphoinositide 3-kinase (PI3K) or Akt (protein kinase B) is a crucial pathway that is aberrantly activated in several types of cancer [9,10,11,12] and regulates core oncogenic processes, such as cell survival, metabolic function, and dissemination [9]. Capivasertib (AZD5363) is a novel selective ATP-competitive isoform of Akt kinase inhibitor that induces comparable effects on all three Akt subtypes: *Akt1*, *Akt2*, and *Akt3* [13,14]. Compared to other AKT inhibitors, capivasertib belongs to the class of ATP-competitive inhibitors that act in competition with adenosine triphosphate (ATP) to bind to Akt kinase at the ATP-binding site of the enzyme. However, P-glycoprotein (Pgp), which consumes ATP, is also regulated by cells. Improved oxidative phosphorylation (OXPHOS), which is associated with adequate ATP production, results in reactive oxygen species (ROS) generation. ROS damages cells by inducing oxidative degradation products or oxidized nucleosides, which have cytotoxic effects [15]. Recent studies have demonstrated that Akt inhibitors (perifosine) precisely sensitize P-glycoprotein-overexpressing drug-resistant breast cancer cells [16]. Furthermore, to maintain hemostatic balance and oxidative stress, metabolism shifts from oxidative phosphorylation to glycolysis, a process mediated by hypoxia-inducible factor 1 alpha (HIF-1α). Under hypoxic or stressful conditions, HIF-1α modulates the transcription of genes encoding enzymes of the glycolytic pathway and glucose transporters, thereby boosting glycolysis to sustain ATP production when oxygen levels are limited [17,18]. This adaptive metabolic switch helps minimize further oxidative stress arising from mitochondrial respiration [19]. Different studies have already proven that HIF-1 is not part of the direct Akt signaling cascade, but that HIF-1 isoform stabilization is mediated via Akt [20,21]. Furthermore, Triciribine (TCN), a selective Akt inhibitor, significantly reduced the expression of phosphorylated Akt, HIF–1α, and survivin in human gastric cancer cells under hypoxic conditions [22]. In contrast, allosteric inhibitors targeting the pleckstrin homology (PH) domain of PI3K prevent its translocation to the plasma membrane for kinase-mediated activation, thereby maintaining Akt in an inactive state [23].

Combination chemotherapy has been used for more than 50 years [24]. The combination of anticancer drugs can overcome resistance and provide new therapeutic options [25]. Since mitochondria play crucial roles in cancer metabolism, regulating the generation of reactive oxygen species (ROS), bioenergetics, and apoptosis, targeting mitochondria in cancer cells is a promising therapeutic strategy. Several approaches have been developed and are currently being investigated to exploit mitochondrial vulnerabilities in cancer treatment [26,27]. For germline *BRCA*-mutated (g*BRCA*m) HER2-negative metastatic breast cancer, targeting mitochondrial functions offers a promising strategy to overcome therapy resistance, which limits the efficacy of conventional treatments, including chemotherapy and PARP inhibitors [28]. Recent research suggests that sporadic tumor-suppressed cellular detachments of *BRCA*2 protein levels are due to mitochondrial DNA (mtDNA) mutations, which may also be responsive to PARP inhibitors [29]. Previous studies have shown that inhibition of PI3K sensitizes *BRCA*-proficient TNBC cell lines, including MDA-MB-231 cells, to PARP inhibition by downregulating *BRCA* expression and inducing DNA damage [9,30]. Therefore, this study aimed to investigate whether the combined treatment of olaparib and capivasertib enhances cytotoxic and anti-clonogenic effects by altering cellular energy production, mitochondrial metabolic function, and reactive oxygen species generation in breast cancer cells.

## 2. Materials and Methods

### 2.1. Drugs

The PARP inhibitor olaparib (AstraZeneca, Redwood City, CA, USA). The Akt inhibitor capivasertib (AZD 5363) was purchased from (Cayman Chemical, Ann Arbor, MI, USA) and dissolved in dimethyl sulfoxide (DMSO) to prepare 10 mM stock solutions. For every experiment, fresh working solutions were prepared by diluting the stock solutions in the culture medium before use.

### 2.2. Cell Cultures

TNBC (MDA-MB-231) and non-TNBC (MCF7) cell lines were purchased from the American Type Culture Collection (ATCC) (Manassas, VA, USA). MDA-MB-231 cells were subjected to culture in Dulbecco’s Modified Eagle Medium (DMEM) with a low glucose concentration, while MCF7 cells were cultured in Roswell Park Memorial Institute 1640 medium (RPMI 1640); both media were prepared with the addition of 10% fetal bovine serum (FBS). Culture medium and FBS were purchased from (Biosera, Cholet, France).

### 2.3. Viability Assay

MCF7 and MDA-MB-231 cells were counted and seeded at a density of 3 × 10^3^ cells per well in 96-well cell culture plates for 24 h before treatment to allow the cells to adhere to the bottom of the plate, reaching approximately 50–60% confluence at the start of treatment in accordance with ATCC recommendations for maintaining cells in logarithmic growth. On the day of the experiment, we treated the cells with 5 µM olaparib, 1 µM capivasertib, and their combination for 72 h. Post-treatment, we applied a viability assay and used the SRB assay according to the following protocol with some modifications [31]. The medium was aspirated, and the cells were washed twice with cold phosphate-buffered saline (PBS) (BIOSERA, Cholet, France). We dispensed 100 µL of cold 10% trichloroacetic acid (TCA) solution (Sigma-Aldrich Co., Budapest, Hungary) into each well, and the plates were kept at 4 °C for 30 min. After incubation, TCA was removed, and the cell culture plates were washed with 1% glacial acetic acid solution (Sigma-Aldrich Co., Budapest, Hungary). The culture plates were then dried overnight at room temperature (RT). After twenty-four hours, a total of 70 microliters of the 0.1% sulforhodamine B (SRB) (Sigma-Aldrich Co., Budapest, Hungary) in 1%glacial acetic acid solution was gently mixed, added to each well, and incubated for 20 min at RT. The culture cell plates were subjected to five rounds of washing with freshly prepared 1% acetic acid solution and air-dried overnight at RT. Next, 200 µL of 10 mM non-neutralized TRIS solution (Sigma-Aldrich Co., Budapest, Hungary) was added to the culture plates, which were subsequently incubated at room temperature on a plate shaker (Biosan SIA, Riga, Latvia) for 3 h. Measurements were taken at 560 nm and 600 nm, one after another, using a GloMax^®^-Multi Detection System (Promega Corporation, Madison, WI, USA). The actual amount of SRB bound to the cellular proteins was calculated by subtracting the optical density (OD)600 from the (OD)560.

### 2.4. Colony Formation Assay

To evaluate the ability of single cells to form colonies during treatment, we seeded the cells at a starting density of 200 cells per 2 mL into 6-well plates and incubated them for 24 h. The next day, the old medium was removed, MCF7 and MDA-MB-231 cells were treated with 5 µM olaparib, 1 µM capivasertib, and their combination for 12 days. The medium was then aspirated, and the cells were washed with PBS and stained with 0.5 g Coomassie Brilliant Blue R stain (Sigma-Aldrich Co., Budapest, Hungary) in 42.86% distilled water (D.W.), 14.29% glacial acetic acid (VWR International, Leuven, Belgium), and 42.86% propanol (E. Merck, Darmstadt, Germany). The culture plates were counted and visualized using a GE Healthcare Image Scanner II (AP Hungary Co., Budapest, Hungary).

### 2.5. Intracellular ROS Production Assay

To quantify the amount of intracellular ROS produced by MCF7 and MDA-MB-231 cells after treatment, the cells were seeded at a density of 3 × 10^3^ cells per well into 96-well plates and incubated for 24 h. Twenty-four hours post-seeding, the cells were exposed to 5 µM olaparib, 1 µM capivasertib, and treated with the drug combination for 72 h. The cells were incubated with 2 µM (DHR123) (Sigma-Aldrich LLC, St. Louis, MO, USA) in a medium supplemented with 10% FBS (Biosera, Cholet, France). The fluorescence intensity of ROS generated from 0 to 2 h was quantified using the fluorescence detection protocol in the GloMax^®^-Multi Detection System (Promega Corporation, Madison, WI, USA) at an excitation wavelength of 490 nm and an emission wavelength of 520 nm.

### 2.6. Apoptosis Assay

To determine the effect of the treatments on MCF7 and MDA-MB-231 cells to induce apoptotic death, a flow cytometer Cell sorter (SONY Biotechnology, San Jose, CA, USA) was used to analyze and determine the ratios of living, early, and late apoptotic cell populations. The cells were seeded into 6-well tissue culture plates at a density of 10^5^ cells per well 24 h before treatment with 5 µM olaparib, 1 µM capivasertib, and their combination for 72 h. At the end of the treatment period, the cells were washed twice with cold BioLegend cell staining buffer using an Eppendorf centrifuge (Eppendorf AG, Hamburg, Germany) at 500× *g* for 5 min at 4 °C. The cells were then resuspended in 100 µL Annexin V Binding Buffer, followed by staining with (5 µL) of fluorochrome-conjugated Annexin V and (10 µL) Propidium Iodide (FITC-Annexin V Apoptosis Detection Kit with PI) (BioLegend, San Diego, CA, USA). After 20 min of incubation at RT and light protection, the samples were suspended in 400 µL of Annexin V Binding buffer before flow cytometric analysis.

### 2.7. Measurement of Bioenergetics

To evaluate the effects of therapeutic agents on MCF7 and MDA-MB-231 cell lines and their impact on energy production, mitochondrial metabolic parameters, including mitochondrial oxidative metabolism and anaerobic energy metabolism, were assessed by measuring OCR and ECAR ratios. For this purpose, the Seahorse XFp Extracellular Flux Analyzer (Agilent Technologies, Santa Clara, CA, USA) was used. Cells were prepared and seeded at an initial density of 3 × 10^3^ cells per well into Seahorse XFp Miniplates for 24 h. The next day, the cells were exposed to 5 µM olaparib, 1 µM capivasterib, and the combination of treatment for 72 h. On the day before the assay, Seahorse XF sensor cartridges were hydrated with Seahorse XF Calibrant and incubated overnight at 37 °C in a non-CO_2_ incubator. This was performed in accordance with the manufacturer’s protocol. The cells were washed twice with the Seahorse XF assay medium, which was prepared according to the protocol of the XFp Mito Stress Test Kit (Agilent Technologies, Santa Clara, CA, USA). Then, 180 µL of medium was added to each well before performing the assay. To detect the core mitochondrial respiratory parameters, the assay was equipped with integrated injection ports on XFp sensor cartridges to dispense the modulators of respiration into the wells. The modulators were injected sequentially in a time-dependent manner. The final concentrations of the modulators were oligomycin (1.5 µM), FCCP (1 µM), and 0.5 µM rotenone and antimycin A. To normalize the data in Wave software, the Micro BCA^TM^ Protein Assay Kit (Thermo Fisher Scientific, Rockford, IL, USA) was used.

### 2.8. Statistical Analysis

Q-Q plots and box plots, along with the Shapiro–Wilk test, were used to evaluate the normality of the data distribution. Levene’s test was used to assess the homogeneity of variances among the groups, ensuring that the presumptions of parametric statistical studies were fulfilled. The findings are shown as the mean ± standard error of the mean (SEM) in three different independent experiments. Statistical analyses were carried out by one-way analysis of variance (ANOVA), followed by Tukey’s post hoc test for multiple comparisons using SPSS version 27 (IBM, Armonk, NY, USA) and GraphPad Prism software version 9 (GraphPad Software, Boston, MA, USA). The statistical significance was set at *p* < 0.05, and each experiment was repeated three times.

## 3. Results

### 3.1. Effects of Single and Combined Olaparib (Op) and Capivasertib (Cap) on Cell Viability

**Figure 1:**
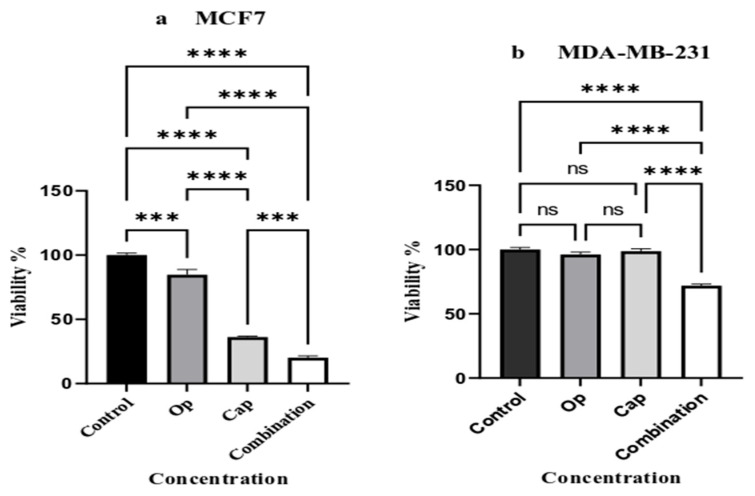
Effect of AKT/PARP inhibitors and their combination on breast cancer cell lines’ viability. MCF7 (**a**) and MDA-MB-231 (**b**) cells were treated with 1 µM capivasertib, 5 µM olaparib, and a combination for 72 h, and cell viability was assessed using the sulforhodamine B (SRB) assay. Data are presented as mean ± standard error of the mean (SEM) of at least three independent experiments performed in triplicate. Statistical significance was determined relative to untreated control cells. Asterisks indicate the level of significance: *** *p* < 0.0002, and **** *p* < 0.00001, respectively, while “ns” indicates no significant difference between groups. Increasing numbers of asterisks correspond to progressively lower *p*-values, indicating a stronger statistical significance.

To assess the anticancer effects, MCF7 (a) and MDA-MB-231 (b) cell lines were treated with 1 µM capivasertib, 5 µM olaparib, and their combination for 72 h, and their viability were evaluated using the sulforhodamine B (SRB) colorimetric method. It was found that MCF7 cell viability was considerably reduced by the treatments (Op and Cap) (Figure 1a). Interestingly, combination therapy decreased the viability of both cell lines significantly (Figure 1a,b).

### 3.2. Effect of Olaparib, Capivasertib, and Their Combination in Colony Formation

**Figure 2:**
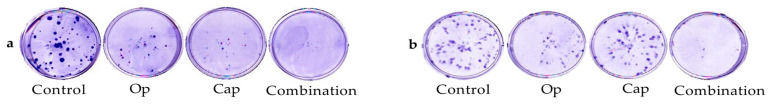

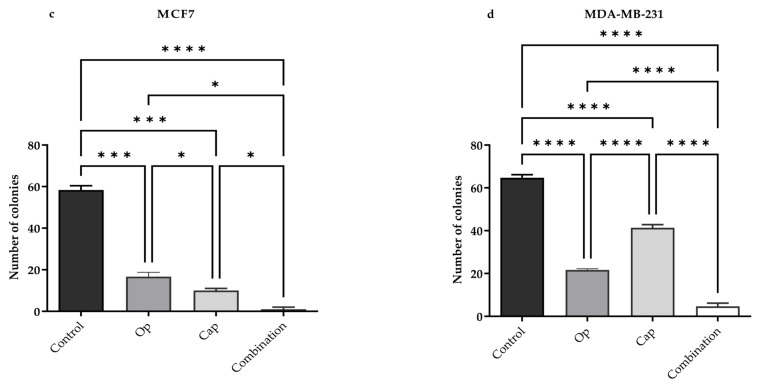
Effect of Olaparib, Capivasertib, and their combination on colony formation of MCF7 (**a**,**c**) and MDA-MB-231 (**b**,**d**) cells. To perform this assay, we exposed the cells to 5 µM Op, 1 µM Cap, and their combinations for 12 days. Colonies were counted, and the results are presented as the mean ± SEM of three independent experiments performed in triplicate. Data was analyzed using one-way analysis of variance (ANOVA), followed by Tukey’s post hoc test for multiple comparisons. Significant differences between groups are indicated as follows: * *p* < 0.05, *** *p* < 0.0010, and **** *p* < 0.00001 compared with control cells (untreated cells).

The colony formation assays were used to demonstrate the capability of cell proliferation and the ability of single cells to form colonies for evaluating the cytostatic effects of PARP and AKT inhibition on human breast cancer cells in HR + BC and TNBC cell lines (Figure 2). Among all treatments, the combination therapy in both cell lines resulted in the most significant reduction in the number of colonies generated (Figure 2).

### 3.3. Effects of Olaparib, Capivasertib, and Their Combination on Reactive Oxygen Species Production in Breast Cancer Cells

**Figure 3:**
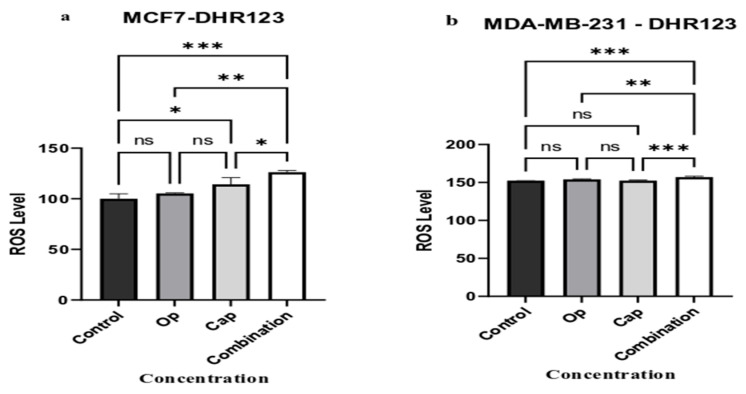
Effects of Olaparib, Capivasertib, and Their Combination on Intracellular ROS Generation in Breast Cancer Cells. MCF7 (**a**) and MDA-MB-231 (**b**) cells were treated with 5 µM Op, 1 µM Cap, and their combination for 72 h. ROS production was assessed post-treatment from the oxidation of non-fluorescent DHR 123. DATA is presented as the mean ± SEM from a minimum of three independent experiments. Significant differences between groups are detailed as follows: * *p* < 0.05, ** *p* < 0.0064, and *** *p* < 0.0004, compared to the control cells, while “ns” indicates no significant differences between groups.

To quantify the level of intracellular ROS, we used DHR 123, providing a sensitive and reliable method based on the fluorescence of rhodamine 123 produced by the oxidation of non-fluorescent DHR 123 by reactive species. Although both cell lines produced ROS upon olaparib exposure, the difference was not statistically significant (Figure 3). However, in MCF7 cells, capivasertib considerably increased ROS production (Figure 3a), whereas in MDA-MB-231 cells, this increase did not reach statistical significance (Figure 3b). Additionally, ROS production was significantly increased in both cell lines following the combination therapy compared to other groups (Figure 3).

### 3.4. Flow Cytometric Analysis of Cell Death Type Induced by Olaparib, Capivasertib, and Their Combination Using Annexin V/Propidium Iodide (PI) Staining

**Figure 4:**
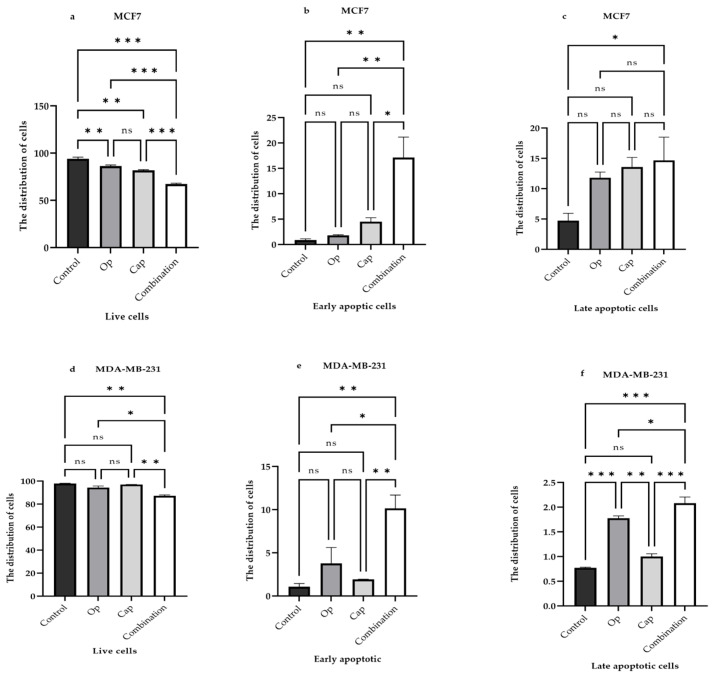

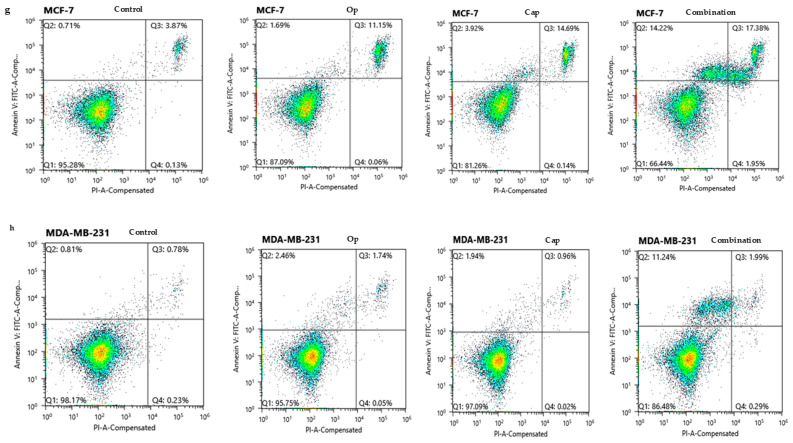
Effect of Op, Cap, and Their Combination on Apoptosis in Breast Cancer Cell Lines. MCF7 (**a**–**c**,**g**) and MDA-MB-231 (**d**–**f**,**h**) cells were treated with 5 µM Op, 1 µM Cap, and their combination for 72 h. Subsequently, the cells were subjected to co-staining with FITC-annexin V and propidium iodide (PI) and analyzed by flow cytometry. In this assay, FITC-annexin V appears green, indicating phosphatidylserine exposure during apoptosis, and propidium iodide appears red, indicating loss of membrane integrity. Plot (**g**,**h**) illustrates the quadrant distribution: Q1, Q2, and Q3 represented live, early, and late (apoptosis), respectively, whereas Q4 represented dead cells. A-Comp indicates the fluorescence channel used for compensation in the flow cytometry. All experiments were performed in triplicate. The results are presented as the mean ± SEM. Significant differences between groups are reported as follows: * *p* < 0.05, ** *p* < 0.0099, and *** *p* < 0.0009, respectively, compared to the control cells. “ns” indicates no significant difference between the groups.

To assess the induction of apoptotic cell death, we treated MCF7 (a, b, c) and MDA-MB-231 (d, e, f) cells with 5 µM Op, 1 µM Cap, and their combination for 72 h. In the MCF7 cell line, the results indicated that olaparib had varying effects on cell distribution. This effect was statistically significant, with a slight reduction in the number of living cells (Figure 4a), whereas in MDA-MB-231 cells, it was statistically significant in the late apoptosis (Figure 4f). Capivasertib did not significantly alter the distribution of MDA-MB-231 cells (Figure 4d–f); however, it dramatically decreased the number of viable MCF7 cells (Figure 4a). In contrast, the combination of the two treatments significantly altered the cell distribution in both cell lines, resulting in a substantial increase in apoptotic cell death (Figure 4).

### 3.5. Effects of Olaparib, Capivasertib, and Their Combinations on Energy Metabolism in Breast Cancer Cells

**Figure 5:**
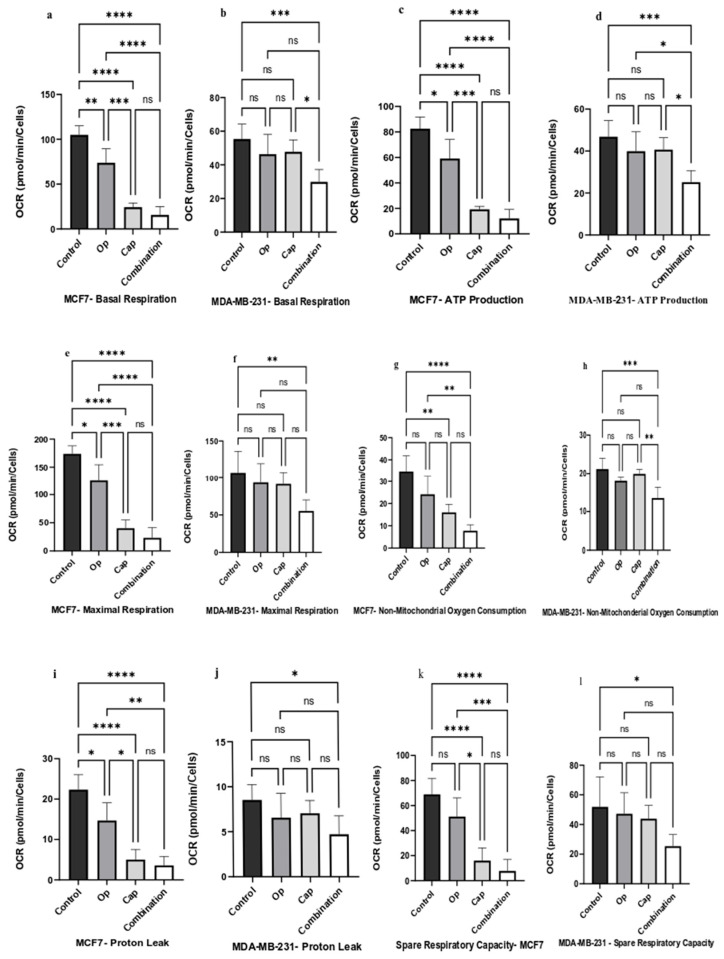
Effect of Op, Cap, and Their Combination on The Energy Metabolism of MCF7 and MDA-MB-231 Cells. Breast cancer cells were treated with 5 µM olaparib, 1 µM capivasertib, and their combination for 72 h. The arrows pointing in both directions indicate (**a**,**b**) basal respiration, (**c**,**d**) ATP production, (**e**,**f**) maximal respiration, (**g**,**h**) non-mitochondrial oxygen consumption, (**i**,**j**) proton leak, and (**k**,**l**) spare respiratory capacity. The results are presented as the mean ± SD of three independent experiments performed in duplicates. Significant differences between groups are reported as follows: * *p* < 0.05, ** *p* < 0.0097, *** *p* < 0.0008, **** *p* < 0.0001, respectively, compared to the control cells. “ns” indicates no significant difference between the groups.

MCF7 and MDA-MB-231 cells were treated with 5 µM Op, 1 µM Cap, and their combination for 72 h, consistent with the duration used in the cell viability assay. Compared to MDA-MB-231 cells, MCF7 cells showed a significant decrease in basal respiration quantification in response to olaparib and capivasertib (Figure 5a,b). However, combination therapy resulted in a considerable decrease in basal respiration among both cell lines (Figure 5a,b). All treatments resulted in a significant decrease in MCF7 cells’ ATP synthesis at different levels (Figure 5c). However, ATP synthesis in MDA-MB-231 cells was significantly affected only by the combination therapy (Figure 5d). The mitochondrial membrane potential disruptor carbonyl cyanide-4 (trifluoromethoxy) phenylhydrazone (FCCP) was injected to measure the maximal cell respiration. The results indicated that maximal cell respiration in MCF7 cells was significantly decreased with all treatments, and the combination treatment showed the largest reduction level in maximal cell respiration (Figure 5e). However, only the combination of drugs in MDA-MB-231 cells showed a significantly decreased level in maximal cell respiration (Figure 5f). Rotenone and antimycin A were used to assess non-mitochondrial respiration by blocking ubiquinone oxidoreductase (complex I) and (complex III) of mitochondrial respiration. Both cell lines exhibited significantly lower non-mitochondrial respiration levels following combination treatment (Figure 5g,h). On the contrary, the human Akt inhibitor (Cap) significantly affected the non-mitochondrial respiration level in MCF7 cells, whereas olaparib (Op) did not affect either of the breast cancer cell lines in the level of non-mitochondrial respiration (Figure 5g,h).

Furthermore, proton leak was considerably decreased in MCF7 cells following treatment with olaparib and capivasertib (Figure 5i), but not to a meaningful extent in MDA-MB-231 cells (Figure 5j). Nonetheless, the degree of proton leakage in both cell lines was significantly decreased following the combination treatment (Figure 5i,j). The difference between maximal and basal respiration was used to measure the spare respiratory capacity. In MCF7 cells, capivasertib significantly decreased spare respiratory capacity (Figure 5k). However, the combination treatment decreased the spare respiratory capacity dramatically in both cell types (Figure 5k,l).

Collectively, these results revealed a novel enhanced effect of combined PARP and AKT inhibition in disrupting mitochondrial bioenergetics in breast cancer cells. Interestingly, while MCF7 cells showed broad sensitivity to monotherapy, marked metabolic damage was observed when combination therapy was used. Both MCF7 and MDA-MB-231 cells suggest a fundamental driver of enhanced vulnerability to disrupt oxidative phosphorylation. This combinatorial treatment uniquely suppresses ATP production, maximal respiration, and spare respiratory capacity while reducing proton leak, highlighting a coordinated shutdown of core mitochondrial functions. These data provide mechanistic insights into how dual PARP and AKT inhibition can overcome metabolic adaptability in breast cancer cells.

### 3.6. Combination of PARP and AKT Inhibition Selectively Impairs Oxidative Phosphorylation in MCF7 and MDA-MB-231 Cell Lines

**Figure 6:**
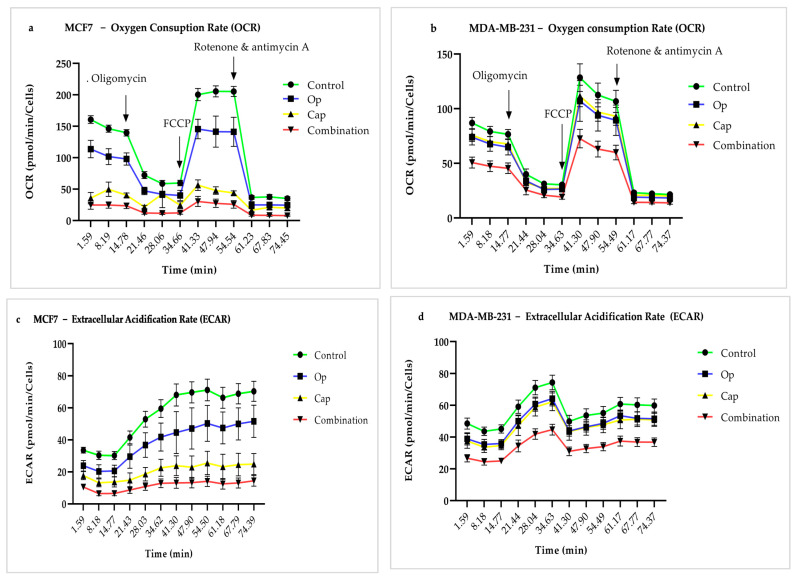

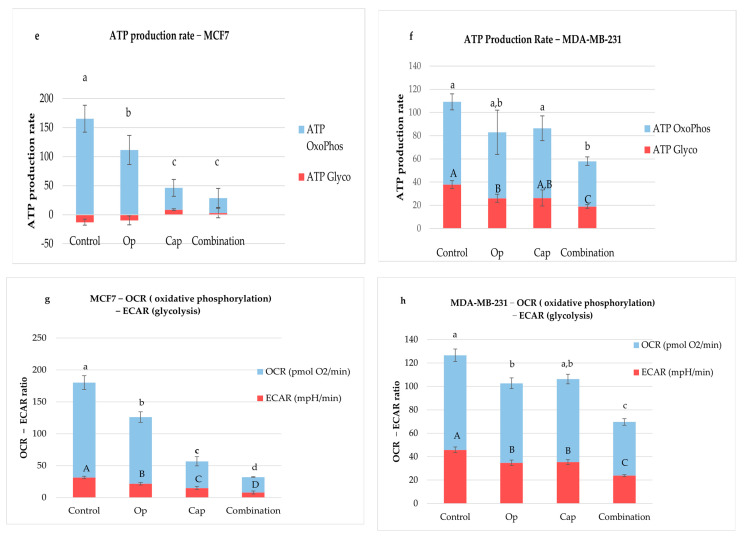
Combined PARP and AKT inhibition disrupted oxidative phosphorylation in breast cancer cells. MCF7 and MDA-MB-231 cells were treated with 5 µM Op, 1 µM Cap, and their combination for 72 h. Oligomycin (O), FCCP, and the respiratory chain inhibitors rotenone (complex I) and antimycin A (complex III) are indicated by the arrows (**a**,**b**). (**a**,**b**) The oxygen consumption rate (OCR) and (**c**,**d**) extracellular acidification rate (ECAR) were determined during the 75 min of the assay measurement. Treatment significantly reduced ATP derived from oxidative phosphorylation (OxPhos) in both cell lines (**e**,**f**), with no significant changes between groups observed in glycolytic ATP production (Glyco) after treatment (**e**,**f**). (**g**,**h**) The metabolic phenotyping, the OCR to ECAR ratio, was calculated from normalized data. Lowercase letters (a, b, c, d) indicate statistically significant differences in oxidative phosphorylation between treatment groups; uppercase letters (A, B, C, D) show the main difference in glycolysis between groups. *p* < 0.05 was considered significant.

To investigate the metabolic effects of PARP and AKT inhibition, MCF7 and MDA-MB-231 cells were treated with 5 µM Op, 1 µM Cap, and their combination for 72 h. Seahorse technology was used to quantify the OCR (Figure 6a,b) and ECAR (Figure 6c,d) during the assay (75 min), and the results indicated that after combined treatment, MCF7 and MDA-MB-231 cells had a higher reduction rate in both ratios compared to monotherapy. The ATP production rate between oxidative phosphorylation and glycolysis was shown in both cell lines, the combination therapy resulted in a significant alteration in ATP production rate during oxidative phosphorylation (OxPhos) compared to other treatments (Figure 6e,f), whereas, glycolytic ATP production (Glyco) was not significantly different among the groups across all conditions in both cell lines (Figure 6e,f).

These results were further supported by metabolic phenotyping through the OCR to ECAR ratio calculation from the normalized data (Figure 6g,h). In both cell lines, the combination treatment showed a trend toward diminished energy production, which was confirmed by a decrease in the oxidative phosphorylation ratio and an absence of a compensatory rise in glycolysis (Figure 6g,h). Overall, these results suggest that the combination of capivasertib and olaparib selectively impaired mitochondrial energy metabolism in breast cancer cell lines without inducing a compensatory glycolytic mechanism. Due to the above-mentioned metabolic impairment effects, dual PARP and AKT inhibition may exert a greater anti-neoplastic impact, suggesting a possible therapeutic option in oncogenic subpopulations with impaired metabolism.

## 4. Discussion

The present study demonstrated that the combination of a human PARP inhibitor (olaparib) and an Akt inhibitor (capivasertib) significantly impaired mitochondrial oxidative phosphorylation in both (MCF7) and (MDA-MB-231) breast cancer cell lines without inducing compensatory glycolytic activation (Figure 6), thereby generating a considerable reduction in overall ATP production in both cell lines (Figure 5c,d). This energy crisis may lead to ROS generation (Figure 3a,b), promote apoptotic cell death (Figure 4), and prevent the colony-forming ability of cells during long-term treatment (Figure 2).

Mitochondria are important targets for cancer therapy [32] because they play critical roles in cancer cell metabolism, survival, and drug resistance [33]. A recent study revealed that intracellular glycolytic processes are remodeled to support mitochondrial oxidative phosphorylation (OXPHOS), thereby sustaining survival, as the glycolytic pathway is downregulated in several cancerous cells of solid tumors. Specific essential amino acids, including glutamate and glutamine, are critical for metabolic reprogramming [34], making the mitochondria a key target. By targeting mitochondrial processes, such as mitochondrial translation, OXPHOS complexes, and mitochondrial membrane permeability, therapeutic strategies can induce cancer cell death and overcome drug resistance [35]. By increasing its activity and PARylation, glutamine deprivation triggers the deoxyribonucleic acid (DNA) damage response and activates PARP-1, a crucial protein involved in DNA repair. By blocking this activation under glutamine deprivation, PARP inhibitors (such as olaparib) increase the susceptibility of cancer cells to DNA damage and decrease their ability to survive [36]. L-glutamine flux coordinates cell growth and proliferation by regulating the translation, autophagy, and mTOR pathways [37]. We studied the rate of ATP synthesis via oxidative phosphorylation (ATP Ox. Phos.) and glycolysis (ATP Glyco). We found that most displayed noticeably more ATP from oxidative phosphorylation; however, there was no discernible difference in ATP production from glycolysis. These results suggested that pyruvate and glutamine in DMEM promoted oxidative phosphorylation in both cell lines (Figure 6).

The current study showed an enhanced cytotoxic effect of the combination of human PARP inhibitors (olaparib) and human Akt inhibitors (capivasertib) in TNBC breast cancer cell line models and MCF7 cells (Figure 1a,b). We evaluated the ability of these compounds to reduce cancer cell viability following short-term treatment. The present study showed that MCF7 cells responded to all treatments, with the most significant reduction in cell number observed with combination therapy (Figure 1a). In contrast, the combination treatment in MDA-MB-231 cells resulted in a significant reduction in the number of cells (Figure 1b). However, the combination treatment impaired the ability of single cells to proliferate and form colonies in both cell lines, and MDA-MB-231 cells reacted more efficiently to the long-term treatment (Figure 2b). We suggest that the combination of olaparib and capivasertib is a promising and effective therapy for reducing tumorigenesis in TNBC cells. As observed during the study, cell growth in combination therapy decreased at a higher rate compared to other treatments, and reduced pyruvate and glutamine in the microenvironment could increase the effectiveness of the treatment.

As demonstrated in the present study, olaparib significantly elevated ROS production in MCF7 cells (Figure 3a), whereas the combination of treatments enhanced intracellular ROS production in both cell lines (Figure 3a,b). Several studies have demonstrated that nicotinamide adenine dinucleotide (NAD^+)^ is an essential metabolite for intracellular homeostatic balance [38,39,40]. Some oxidation–reduction reactions are dependent on NAD^+^ to obtain energy. NAD^+^ serves as a substrate for sirtuins, PARPs, and cyclic ADP-ribose(s) cADPRs in multiple signaling pathways, and the NAD^+^ pool has been associated with tumorigenesis. Cancerous cells switch from normal oxidative phosphorylation to glycolytic metabolism to generate a rapid ATP biosynthetic rate, regulate redox balance, and synthesize nucleic acids, proteins, and fatty acids [40]. This metabolic remodeling, also known as the Warburg effect, promotes tumor growth and cancer progression [41]. Conversely, impairment of oxidative phosphorylation can lead to a reduction in mitochondrial β-oxidation in treatment-resistant estrogen receptor-positive breast cancer (ER + BC) patients [42].

A recent study demonstrated that PARP inhibitors lead to increased oxidative stress in cancer cells and may enhance their sensitivity of cancer cells to hydrogen peroxide (H_2_O_2_), leading to increased cytotoxicity, inhibition of PARP, and triggering oxidative DNA damage in neoplastic cells [43].

Innovative research has indicated that increased ROS production in MDA-MB-231 and MCF-7 cells induces apoptosis [44]. ATPase inhibitory factor 1 (IF1) has been reported to influence mitochondrial function and may contribute to cytoprotective responses, including the regulation of inflammation, oxidative stress, and cell survival. H^+^-ATP synthase, an essential component of oxidative phosphorylation, plays a crucial role in maintaining cellular metabolic and redox balance [45]. Suppression of Akt and concurrent AMPK activity may change cell signaling from energy consumption via Akt activation to energy metabolic production regulated by AMPK activation, thus compensating for energy depletion under stressful conditions [46].

To overcome drug resistance induced in TNBC and maintain energy at the minimal limit, we applied double-downstream signaling of Akt together with PARP inhibition. We used the Agilent Seahorse XFp Cell Mito Test to evaluate differences between ATP production from oxidative phosphorylation and glycolysis. On the one hand, we found that olaparib, capivasertib, and the combination of treatments reduced the metabolic activity of both cell lines by decreasing oxidative phosphorylation without inducing compensatory glycolytic activation (Figure 6g,h), resulting in a marked decline in overall ATP production (Figure 5). Notably, neither monotherapy nor combination treatment resulted in increased glycolytic ATP production, indicating that these cells did not compensate for metabolic changes by switching to glycolysis under stress (Figure 6e,f).

However, the effect of capivasertib and olaparib, alone or in combination, on the phosphorylation levels of p66 ShcA S36 remains unclear. The existing literature explains their role in electron transfer to oxidizing cytochrome c to induce ROS production, which is crucial for apoptotic signaling [47]. Mitochondria, which are central to apoptosis, induces the release of pro-apoptotic factors, such as cytochrome c, when membrane integrity is impaired, often stimulated by the permeability transition pore (PTP) [48,49,50]. Additionally, continuous damage to complex I worsens heart injury during reperfusion by interfering with energy production, encouraging the formation of reactive oxygen species, and increasing the chances of mitochondrial permeability transition pore opening [51]. Here, we demonstrated that combination treatment reduced non-mitochondrial oxygen consumption significantly better than monotherapy with olaparib and capivasertib (Figure 5g,h). The opening of the mPTP triggers the increased generation and release of mROS, which harms both mitochondrial and nuclear DNA [52].

A recent study showed that combination therapy with olaparib and a third-generation oncolytic adenovirus (TS-2021) was effective against human glioblastoma (GBM) in vivo and in vitro. Additionally, they demonstrated that the effectiveness of this combination therapy relies on DNA damage and apoptotic cell death [53]. In the present study, we investigated whether these treatments induced apoptosis. We performed flow cytometric analysis for cell death and found that capivasertib did not significantly induce apoptotic cell death in either cell line (Figure 4). As previously demonstrated, the experimental Akt inhibitor (LY294002) did not have significant effects on cell distribution in either cell line [54]. In contrast, olaparib showed a statistically significant difference in the distribution of MDA-MB-231 cells, and some dead cells were detected during late-stage apoptosis (Figure 4). In addition, the combination of treatments induced apoptosis in both cell lines, as indicated by the bar graphs, and the combination of treatments had a significant effect on cell distribution (Figure 4). The present study was strengthened by a comprehensive metabolic analysis and consistent findings across two distinct breast cancer cell models. However, this study had several limitations. First, the effects of these treatments on ATP production and fatty acid oxidation in metabolically active tissues, such as the heart and liver, have not been evaluated in vivo. Given the essential role of mitochondrial metabolism in healthy tissues, future studies using animal models are necessary to assess the systemic metabolic impact and safety profile of this combinatorial strategy.

## 5. Conclusions

We concluded that the simultaneous inhibition of PARP and Akt using olaparib and capivasertib triggers metabolic collapse and apoptotic cell death in breast cancer cells, particularly in triple-negative breast cancer (TNBC) and MCF7 cells. This disrupts mitochondrial bioenergetics, increases reactive oxygen species (ROS) levels, and promotes apoptosis. This dual-targeted approach impairs oxidative phosphorylation and suppresses compensation for metabolic changes by switching to glycolysis, particularly under mitochondrial stress. Combining metabolic and DNA repair stress, this strategy offers a promising avenue for overcoming drug resistance in breast cancer cells and selectively inducing cell death via redox-sensitive apoptotic mechanisms. Further studies are necessary to investigate ATP production and fatty acid oxidation in metabolically active tissues in vivo. This will allow for a comprehensive evaluation of the systemic efficacy and safety of combined Akt and PARP inhibition.

## Data Availability

Additional data supporting the study’s findings can be obtained from the corresponding author upon a reasonable request.
